# 1-(4-Chloro­benzo­yl)-3-(3-methyl­pyridin-2-yl)thio­urea

**DOI:** 10.1107/S1600536811032375

**Published:** 2011-08-17

**Authors:** M.Sukeri M. Yusof, Nurwahyuni A. Mushtari, Maisara A. Kadir, Bohari M. Yamin

**Affiliations:** a Department of Chemical Sciences, Faculty of Science and Technology, Universiti Malaysia Terengganu, 21030 Kuala Terengganu, Terengganu, Malaysia; b School of Chemical Sciences and Food Technology, Universiti Kebangsaan Malaysia, UKM 43500 Bangi Selangor, Malaysia

## Abstract

The mol­ecule of the title compound, C_14_H_12_ClN_3_OS, consists of three approximately planar fragments: the central thio­urea group, the chloro­phenyl group and the picolyl (3-methyl­pyridin-2-yl) group with a maximum of 0.035 (2)° for an N atom from the mean square plane of the central thiourea group. The central fragment forms dihedral angles of 33.30 (8) and 76.78 (8)° with the chloro­phenyl and picolyl groups, respectively. With respect to the thio­urea C—N bonds, the 4-chloro­benzoyl group is positioned *trans* to the thiono S atoms, whereas the picolyl group lies in a *cis* position to it. The mol­ecular conformation is stabilized by an intra­molecular N—H⋯O hydrogen bond. In the crystal, mol­ecules are linked by inter­molecular C—H⋯N hydrogen bonds, forming chains along the *a* axis.

## Related literature

For applications of thio­urea derivatives, see: Cunha *et al.* (2007 ▶); Srivastava *et al.* (2010 ▶); Manjula *et al.* (2009 ▶); Chen *et al.* (2006 ▶). For related structures, see: Estévez-Hernández *et al.* (2009 ▶); Binzet *et al.* (2009 ▶). For standard bond lengths, see: Allen *et al.* (1987 ▶).
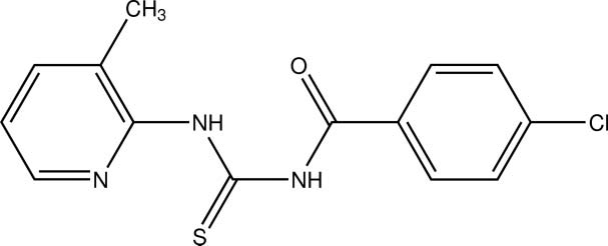


## Experimental

### 

#### Crystal data

C_14_H_12_ClN_3_OS
*M* *_r_* = 305.78
Monoclinic, 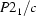

*a* = 7.8417 (15) Å
*b* = 7.1058 (13) Å
*c* = 25.585 (5) Å
β = 93.405 (4)°
*V* = 1423.1 (5) Å^3^
*Z* = 4
Mo *K*α radiation
μ = 0.41 mm^−1^
*T* = 298 K
0.44 × 0.31 × 0.14 mm

#### Data collection

Bruker SMART APEX CCD area-detector diffractometer
Absorption correction: multi-scan (*SADABS*; Bruker, 2003 ▶) *T*_min_ = 0.839, *T*_max_ = 0.944
9917 measured reflections
3421 independent reflections
2188 reflections with *I* > 2/s(*I*)
*R*_int_ = 0.030

#### Refinement

*R*[*F* ^2^ > 2σ(*F* ^2^)] = 0.053
*wR*(*F* ^2^) = 0.139
*S* = 1.04
3421 reflections
181 parameters
H-atom parameters constrained
Δρ_max_ = 0.37 e Å^−3^
Δρ_min_ = −0.17 e Å^−3^

### 

Data collection: *SMART* (Bruker, 2003 ▶); cell refinement: *SAINT* (Bruker, 2003 ▶); data reduction: *SAINT*; program(s) used to solve structure: *SHELXS97* (Sheldrick, 2008 ▶); program(s) used to refine structure: *SHELXL97* (Sheldrick, 2008 ▶); molecular graphics: *SHELXTL* (Sheldrick, 2008 ▶); software used to prepare material for publication: *SHELXTL*, *PARST* (Nardelli, 1995 ▶) and *PLATON* (Spek, 2009 ▶).

## Supplementary Material

Crystal structure: contains datablock(s) global, I. DOI: 10.1107/S1600536811032375/yk2015sup1.cif

Structure factors: contains datablock(s) I. DOI: 10.1107/S1600536811032375/yk2015Isup2.hkl

Supplementary material file. DOI: 10.1107/S1600536811032375/yk2015Isup3.cml

Additional supplementary materials:  crystallographic information; 3D view; checkCIF report

## Figures and Tables

**Table 1:** Hydrogen-bond geometry (Å, °)

*D*—H⋯*A*	*D*—H	H⋯*A*	*D*⋯*A*	*D*—H⋯*A*
N2—H2*A*⋯O1	0.86	1.98	2.655 (2)	135
C2—H2*B*⋯N3^i^	0.93	2.59	3.417 (3)	148

Symmetry code: (i) 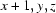.

**Fig. 1.**
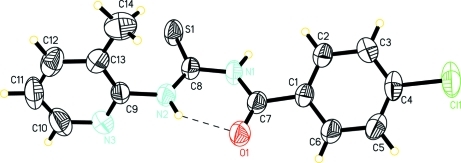
The molecular structure of (I), with displacement ellipsods drawn at the 50% probability level.

**Fig. 2.**
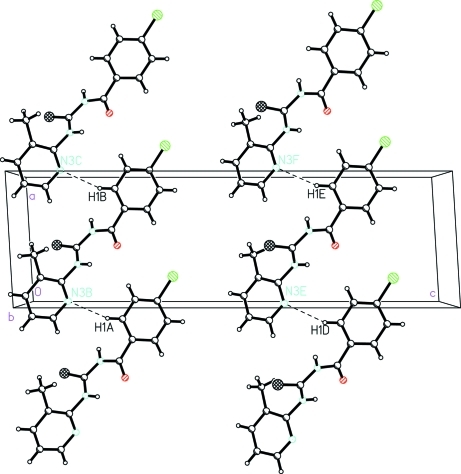
A packing diagram of (I) viewed down the b axis. Hydrogen bonds are shown by dashed lines.

## supplementary crystallographic information

### Comment

The synthesis of new thiourea derivatives has attracted great interest because of their wide range applications in research and technology, such as in pharmacology (Cunha *et al.*, 2007), catalysis (Chen *et al.*, 2006) and agriculture (Srivastava *et al.*, 2010). The title compound, (I), is an isomer of the previously reported compound, 4-chloro-*N*-[*N*-(6-methyl-2-pyridyl)-carbamothioyl]benzamide (Binzet *et al.*, 2009) except the methyl group is attached at the third position of the pyridine ring. The molecule adopts *trans-cis* configuration with respect to the position of 4-chlorobenzoyl and 2-picolyl groups relative to the thiono S atom across the thiourea C—N bonds. The bond lengths and angles are within normal ranges (Allen *et al.*, 1987) and agree with previously reported analogous molecules (Estévez-Hernández *et al.*, 2009; Binzet *et al.*, 2009). The molecule consists of central thiourea fragment (N2/C8/S1/N1), pyridine (C9—C13/N3) and phenyl (C1—C6) rings which are nearly planar with largest deviation from the least square plane of 0.035 (2)Å for N1 atom.

The molecule is stabilized by intramolecular hydrogen bond, O1···H2A—N2, forming a pseudo-six membered ring, O1···H2A—N2—C8—N1—C7—O1 (Table 1). In crystal the molecules are linked by intermolecular hydrogen bond C2—H2···N3^i^, forming chains along the *a* axis (Fig. 2).

### Experimental

2-amino-3-picoline (1.0 g, 0.5 mmol) was added dropwise into the mixture of ammonium thiocyanate (0.62 g, 0.5 mmol) and 4-chlorobenzoyl chloride (0.44 g, 0.5 mmol) diluted by 50 ml of dry acetone. The reaction mixture was refluxed with permanent stirring for 3 h. The resulting precipitate was filtered off and washed with cold methanol. The colorless crystals were obtained by recrystallization from acetonitrile. Yield: 46%; m.p. 170.1–171.1°C

### Refinement

H atoms on the parent carbon atoms were positioned geometrically with C—H= 0.93–0.96 Å and constrained to ride on their parent atoms with *U*_iso_(H)= *xU*_eq_(parent atom) where *x*=1.5 for CH_3_ group and 1.2 for CH groups.

### Crystal data

**Table:**

C_14_H_12_ClN_3_OS	*F*(000) = 632
*M**_r_* = 305.78	*D*_x_ = 1.427 Mg m^−^^3^
Monoclinic, *P*2_1_/*c*	Mo *K*α radiation, λ = 0.71073 Å
Hall symbol: -P 2ybc	Cell parameters from 699 reflections
*a* = 7.8417 (15) Å	θ = 1.6–28.0°
*b* = 7.1058 (13) Å	µ = 0.41 mm^−^^1^
*c* = 25.585 (5) Å	*T* = 298 K
β = 93.405 (4)°	Slab, colourless
*V* = 1423.1 (5) Å^3^	0.44 × 0.31 × 0.14 mm
*Z* = 4	

### Data collection

**Table:**

Bruker SMART APEX CCD area-detector diffractometer	3421 independent reflections
Radiation source: fine-focus sealed tube	2188 reflections with *I* > 2/s(*I*)
graphite	*R*_int_ = 0.030
Detector resolution: 83.66 pixels mm^-1^	θ_max_ = 28.0°, θ_min_ = 1.6°
ω scans	*h* = −10→10
Absorption correction: multi-scan (*SADABS*; Bruker, 2003)	*k* = −9→9
*T*_min_ = 0.839, *T*_max_ = 0.944	*l* = −30→33
9917 measured reflections	

### Refinement

**Table:**

Refinement on *F*^2^	Primary atom site location: structure-invariant direct methods
Least-squares matrix: full	Secondary atom site location: difference Fourier map
*R*[*F*^2^ > 2σ(*F*^2^)] = 0.053	Hydrogen site location: inferred from neighbouring sites
*wR*(*F*^2^) = 0.139	H-atom parameters constrained
*S* = 1.04	*w* = 1/[σ^2^(*F*_o_^2^) + (0.0722*P*)^2^ + 0.0755*P*] where *P* = (*F*_o_^2^ + 2*F*_c_^2^)/3
3421 reflections	(Δ/σ)_max_ = 0.001
181 parameters	Δρ_max_ = 0.37 e Å^−^^3^
0 restraints	Δρ_min_ = −0.17 e Å^−^^3^

### Special details

**Table:**

Geometry. All e.s.d.'s (except the e.s.d. in the dihedral angle between two l.s. planes) are estimated using the full covariance matrix. The cell e.s.d.'s are taken into account individually in the estimation of e.s.d.'s in distances, angles and torsion angles; correlations between e.s.d.'s in cell parameters are only used when they are defined by crystal symmetry. An approximate (isotropic) treatment of cell e.s.d.'s is used for estimating e.s.d.'s involving l.s. planes.
Refinement. Refinement of *F*^2^ against ALL reflections. The weighted *R*-factor *wR* and goodness of fit *S* are based on *F*^2^, conventional *R*-factors *R* are based on *F*, with *F* set to zero for negative *F*^2^. The threshold expression of *F*^2^ > σ(*F*^2^) is used only for calculating *R*-factors(gt) *etc*. and is not relevant to the choice of reflections for refinement. *R*-factors based on *F*^2^ are statistically about twice as large as those based on *F*, and *R*- factors based on ALL data will be even larger.

### Fractional atomic coordinates and isotropic or equivalent isotropic displacement parameters (Å^2^)

**Table:**

	*x*	*y*	*z*	*U*_iso_*/*U*_eq_	
Cl1	1.21606 (9)	0.54075 (13)	0.35489 (3)	0.0897 (3)	
S1	0.45565 (8)	0.34425 (9)	0.08001 (2)	0.0597 (2)	
O1	0.4546 (2)	0.6680 (3)	0.23221 (6)	0.0657 (5)	
N1	0.5639 (2)	0.4758 (3)	0.17178 (7)	0.0496 (5)	
H1A	0.6480	0.4014	0.1669	0.060*	
N2	0.3126 (2)	0.6067 (3)	0.13694 (7)	0.0501 (5)	
H2A	0.3116	0.6688	0.1658	0.060*	
N3	0.0289 (2)	0.5707 (3)	0.10740 (8)	0.0604 (5)	
C2	0.8891 (3)	0.5112 (3)	0.23027 (9)	0.0485 (5)	
H2B	0.8932	0.4886	0.1946	0.058*	
C3	1.0381 (3)	0.5047 (3)	0.26218 (10)	0.0537 (6)	
H3A	1.1421	0.4772	0.2483	0.064*	
C4	1.0291 (3)	0.5398 (3)	0.31489 (9)	0.0552 (6)	
C5	0.8769 (3)	0.5791 (3)	0.33639 (9)	0.0582 (6)	
H5A	0.8734	0.6016	0.3721	0.070*	
C6	0.7299 (3)	0.5850 (3)	0.30452 (8)	0.0537 (6)	
H6A	0.6264	0.6120	0.3188	0.064*	
C1	0.7344 (3)	0.5511 (3)	0.25111 (8)	0.0429 (5)	
C7	0.5720 (3)	0.5711 (3)	0.21855 (8)	0.0465 (5)	
C8	0.4375 (3)	0.4836 (3)	0.13118 (8)	0.0446 (5)	
C9	0.1793 (3)	0.6406 (3)	0.09725 (8)	0.0437 (5)	
C10	−0.0985 (3)	0.6033 (4)	0.07152 (12)	0.0758 (8)	
H10A	−0.2062	0.5565	0.0777	0.091*	
C11	−0.0802 (4)	0.7006 (4)	0.02671 (12)	0.0784 (9)	
H11A	−0.1723	0.7180	0.0026	0.094*	
C12	0.0767 (4)	0.7723 (4)	0.01786 (10)	0.0689 (7)	
H12A	0.0919	0.8398	−0.0127	0.083*	
C13	0.2135 (3)	0.7461 (3)	0.05369 (8)	0.0508 (5)	
C14	0.3859 (4)	0.8282 (4)	0.04571 (12)	0.0781 (8)	
H14A	0.3823	0.8960	0.0132	0.117*	
H14B	0.4686	0.7289	0.0448	0.117*	
H14C	0.4172	0.9125	0.0740	0.117*	

### Atomic displacement parameters (Å^2^)

**Table:**

	*U*^11^	*U*^22^	*U*^33^	*U*^12^	*U*^13^	*U*^23^
Cl1	0.0655 (5)	0.1158 (7)	0.0829 (5)	−0.0260 (4)	−0.0364 (4)	0.0270 (4)
S1	0.0599 (4)	0.0634 (4)	0.0533 (4)	0.0146 (3)	−0.0178 (3)	−0.0192 (3)
O1	0.0578 (10)	0.0846 (13)	0.0529 (10)	0.0209 (9)	−0.0111 (8)	−0.0216 (9)
N1	0.0472 (10)	0.0533 (11)	0.0462 (10)	0.0133 (8)	−0.0145 (8)	−0.0115 (8)
N2	0.0474 (10)	0.0602 (12)	0.0416 (10)	0.0094 (9)	−0.0065 (8)	−0.0091 (8)
N3	0.0439 (11)	0.0756 (14)	0.0614 (12)	0.0035 (10)	0.0013 (9)	0.0011 (10)
C2	0.0529 (13)	0.0471 (13)	0.0443 (12)	−0.0004 (10)	−0.0076 (10)	−0.0045 (9)
C3	0.0443 (13)	0.0532 (14)	0.0629 (15)	−0.0014 (10)	−0.0043 (11)	0.0023 (11)
C4	0.0547 (14)	0.0499 (14)	0.0579 (14)	−0.0154 (11)	−0.0227 (12)	0.0120 (11)
C5	0.0666 (16)	0.0654 (16)	0.0411 (12)	−0.0146 (13)	−0.0087 (11)	0.0011 (11)
C6	0.0521 (13)	0.0615 (15)	0.0467 (13)	−0.0054 (11)	−0.0057 (10)	−0.0036 (11)
C1	0.0483 (12)	0.0382 (11)	0.0408 (11)	−0.0016 (9)	−0.0078 (9)	−0.0026 (9)
C7	0.0478 (12)	0.0487 (13)	0.0423 (12)	0.0026 (10)	−0.0046 (10)	−0.0046 (10)
C8	0.0431 (12)	0.0467 (12)	0.0429 (11)	0.0010 (9)	−0.0068 (9)	−0.0021 (9)
C9	0.0426 (12)	0.0454 (12)	0.0422 (11)	0.0077 (9)	−0.0047 (9)	−0.0048 (9)
C10	0.0441 (14)	0.092 (2)	0.090 (2)	0.0071 (14)	−0.0097 (14)	−0.0119 (18)
C11	0.074 (2)	0.083 (2)	0.0737 (19)	0.0307 (16)	−0.0323 (15)	−0.0139 (16)
C12	0.096 (2)	0.0565 (16)	0.0528 (15)	0.0230 (15)	−0.0098 (14)	0.0027 (12)
C13	0.0644 (15)	0.0406 (12)	0.0471 (12)	0.0067 (11)	0.0003 (11)	−0.0024 (10)
C14	0.088 (2)	0.0666 (18)	0.0811 (19)	−0.0112 (15)	0.0187 (16)	0.0070 (15)

### Geometric parameters (Å, °)

**Table:**

Cl1—C4	1.738 (2)	C5—C6	1.372 (3)
S1—C8	1.654 (2)	C5—H5A	0.9300
O1—C7	1.218 (3)	C6—C1	1.390 (3)
N1—C7	1.373 (3)	C6—H6A	0.9300
N1—C8	1.394 (2)	C1—C7	1.487 (3)
N1—H1A	0.8600	C9—C13	1.382 (3)
N2—C8	1.328 (3)	C10—C11	1.354 (4)
N2—C9	1.434 (3)	C10—H10A	0.9300
N2—H2A	0.8600	C11—C12	1.363 (4)
N3—C9	1.320 (3)	C11—H11A	0.9300
N3—C10	1.337 (3)	C12—C13	1.382 (3)
C2—C3	1.385 (3)	C12—H12A	0.9300
C2—C1	1.383 (3)	C13—C14	1.498 (4)
C2—H2B	0.9300	C14—H14A	0.9600
C3—C4	1.377 (3)	C14—H14B	0.9600
C3—H3A	0.9300	C14—H14C	0.9600
C4—C5	1.372 (4)		
			
C7—N1—C8	128.59 (18)	O1—C7—C1	122.05 (18)
C7—N1—H1A	115.7	N1—C7—C1	115.77 (19)
C8—N1—H1A	115.7	N2—C8—N1	116.12 (18)
C8—N2—C9	122.93 (17)	N2—C8—S1	125.52 (16)
C8—N2—H2A	118.5	N1—C8—S1	118.34 (15)
C9—N2—H2A	118.5	N3—C9—C13	125.6 (2)
C9—N3—C10	116.1 (2)	N3—C9—N2	114.81 (19)
C3—C2—C1	120.5 (2)	C13—C9—N2	119.6 (2)
C3—C2—H2B	119.7	N3—C10—C11	123.9 (3)
C1—C2—H2B	119.7	N3—C10—H10A	118.1
C4—C3—C2	118.7 (2)	C11—C10—H10A	118.1
C4—C3—H3A	120.6	C10—C11—C12	118.3 (3)
C2—C3—H3A	120.6	C10—C11—H11A	120.9
C3—C4—C5	121.7 (2)	C12—C11—H11A	120.9
C3—C4—Cl1	119.1 (2)	C11—C12—C13	120.8 (2)
C5—C4—Cl1	119.12 (19)	C11—C12—H12A	119.6
C6—C5—C4	119.2 (2)	C13—C12—H12A	119.6
C6—C5—H5A	120.4	C9—C13—C12	115.3 (2)
C4—C5—H5A	120.4	C9—C13—C14	122.8 (2)
C5—C6—C1	120.6 (2)	C12—C13—C14	121.9 (2)
C5—C6—H6A	119.7	C13—C14—H14A	109.5
C1—C6—H6A	119.7	C13—C14—H14B	109.5
C6—C1—C2	119.2 (2)	H14A—C14—H14B	109.5
C6—C1—C7	117.6 (2)	C13—C14—H14C	109.5
C2—C1—C7	123.05 (19)	H14A—C14—H14C	109.5
O1—C7—N1	122.2 (2)	H14B—C14—H14C	109.5

### Hydrogen-bond geometry (Å, °)

**Table:**

*D*—H···*A*	*D*—H	H···*A*	*D*···*A*	*D*—H···*A*
N2—H2A···O1	0.86	1.98	2.655 (2)	135
C2—H2B···N3^i^	0.93	2.59	3.417 (3)	148

Symmetry codes:  (i) *x*+1, *y*, *z*.
